# *Lactobacillus plantarum* DR7 Modulated Bowel Movement and Gut Microbiota Associated with Dopamine and Serotonin Pathways in Stressed Adults

**DOI:** 10.3390/ijms21134608

**Published:** 2020-06-29

**Authors:** Guoxia Liu, Hui-Xian Chong, Fiona Yi-Li Chung, Yin Li, Min-Tze Liong

**Affiliations:** 1 CAS Key Laboratory of Microbial Physiological and Metabolic Engineering, State Key Laboratory of Microbial Resources, Institute of Microbiology, Chinese Academy of Sciences, Beijing 100101, China; liuguoxia@im.ac.cn; 2 School of Industrial Technology, Universiti Sains Malaysia, Penang 11800, Malaysia; huixian1126@gmail.com (H.-X.C.); fionacyl@outlook.com (F.Y.-L.C.)

**Keywords:** *Lactobacillus plantarum*, probiotic, microbiota, stress, serotonin, dopamine, clinical trial

## Abstract

We have previously reported that the administration of *Lactobacillus plantarum* DR7 for 12 weeks reduced stress and anxiety in stressed adults as compared to the placebo group, in association with changes along the brain neurotransmitters pathways of serotonin and dopamine-norepinephrine. We now aim to evaluate the effects of DR7 on gut functions, gut microbiota compositional changes, and determine the correlations between microbiota changes and the pathways of brain neurotransmitters. The administration of DR7 prevented an increase of defecation frequency over 12 weeks as compared to the placebo (*p* = 0.044), modulating the increase of stress-induced bowel movement. Over 12 weeks, alpha diversity of gut microbiota was higher in DR7 than the placebo group across class (*p* = 0.005) and order (*p* = 0.018) levels, while beta diversity differed between groups at class and order levels (*p* < 0.001). Differences in specific bacterial groups were identified, showing consistency at different taxonomic levels that survived multiplicity correction, along the phyla of Bacteroides and Firmicutes and along the classes of Deltaproteobacteria and Actinobacteria. Bacteroidetes, Bacteroidia, and Bacteroidales which were reduced in abundance in the placebo group showed opposing correlation with gene expression of dopamine beta hydrolase (DBH, dopamine pathway; *p* < 0.001), while Bacteroidia and Bacteroidales showed correlation with tryptophan hydroxylase-II (TPH2, serotonin pathway; *p* = 0.001). A correlation was observed between DBH and Firmicutes (*p* = 0.002), Clostridia (*p* < 0.001), Clostridiales (*p* = 0.001), *Blautia* (*p* < 0.001), and *Romboutsia* (*p* < 0.001), which were increased in abundance in the placebo group. *Blautia* was also associated with TDO (*p* = 0.001), whereas *Romboutsia* had an opposing correlation with TPH2 (*p* < 0.001). Deltaproteobacteria and Desulfovibrionales which were decreased in abundance in the placebo group showed opposing correlation with DBH (*p* = 0.001), whereas *Bilophila* was associated with TPH2 (*p* = 0.001). Our present data showed that physiological changes induced by *L. plantarum* DR7 could be associated with changes in specific taxa of the gut microbiota along the serotonin and dopamine pathways.

## 1. Introduction

The symptoms of stress affect human anatomy beyond that of psychological perceptions. Increasing consumer awareness on health have led to better characterization and understanding of stress, on both aetiology and consequences. One of the more vastly reported bodily changes as influenced by stress includes gastrointestinal functions, where symptoms of heartburn, indigestion, nausea and vomiting, bowel movement, and abdominal pain are reportedly increased amid stress. Recent advances in gut microbiota profiling have garnered much evidence that these gut inhabitants play major roles in host physiological signaling and responses, including psychiatric conditions along the gut–brain axis. Mounting evidence suggests that microbial cellular components and metabolites of the complex gut microbiota may influence brain functions via neuroimmune and neuroendocrine pathways as well as the nervous system, while differences in microbial diversity and taxonomic compositions were observed between stressed and control individuals [1]. Although gut microbiota changes with growth, primarily attributed to external factors such as environment, mode of delivery, diet, and lifestyles, dysbiosis also occurs upon changes in health conditions such as bowel disorders and inflammatory and metabolic diseases [2]. It is thus suggested that the modulation of gut microbiota is crucial towards a healthier general well-being, including that of mental health.

Probiotics are “live microorganisms that, when administered in adequate amounts, confer a health benefit on the host” [3]. *Lactobacillus* remains one of the most commonly administered probiotic genera with a long history of safe use and comprehensive documentation on gut health and antimicrobial protective properties [4]. Increasing evidence has shown the potentials of probiotics as a natural agent to influence brain health and psychological well-being. Anxiolytic and antidepressant-like behaviors were observed in mice administered with *Lactobacillus rhamnosus* while exaggerated hypothalamic–pituitary–adrenal stress response in germ-free mice was partially reversed upon oral consumption of *Bifidobacterium infantis* [5]. Although depression-like behaviors in a rat model were reversed upon administration of *Bifidobacterium infantis* [6], studies on the association of major depressive disorders (MDD) with gut microbiota revealed that MDD patients showed a lower abundance of gut *Bifidobacterium* and *Lactobacillus* than healthy controls [7].

We have previously reported on the use of *Lactobacillus plantarum* DR7 (now *Lactoplantibacillus plantarum* DR7 [8]) in the alleviation of stress and anxiety in stressed adults, accompanied by improved traits of memory and cognition such as basic attention, emotional cognition, and associate learning as compared to the placebo group [9]. DR7 was administered in a randomized, double-blind, and placebo-controlled study, where plasma cortisol levels were reduced accompanied by reduced plasma pro-inflammatory cytokines, such as interferon-γ (IFN- γ) and tumor necrosis factor-α (TNF-α), and increased plasma anti-inflammatory cytokines, such as interleukin-10 (IL-10), compared to the placebo. Plasma gene expression analysis revealed that DR7 exerted these psychological effects along the brain neurotransmitters pathway of serotonin synthesis from tryptophan and the dopamine-norepinephrine pathway. The study started during the year-end period of 2017 in East Peninsular Malaysia, which also corresponded to the period of annual monsoon season, for 12 weeks, which coincided with the aftermath of a flood. During the monsoon of 2017, a massive flood occurred in East Peninsular Malaysia, affecting over 14,000 individuals and displacing over 2000 homes where families were housed in shelters [10], attributed to increased rainfall in November and December 2017. We have also previously reported that these monsoon and flood seasons imposed great stress, gut dysbiosis, and abdominal disorders in flood victims and the general communities of East Peninsular Malaysia [11].

Considering the gut–brain axis is bidirectional while probiotics are orally administered to reach the gut, we aimed to evaluate and better understand the effects of DR7 on gut disorders and gut microbiota compositional changes. More importantly, we aimed to determine the potential relationships between microbiota changes and the pathways of brain neurotransmitters that had led to improved psychological effects in stressed subjects as previously reported [9].

## 2. Results and Discussion

### 2.1. Baseline

**Figure 1:**
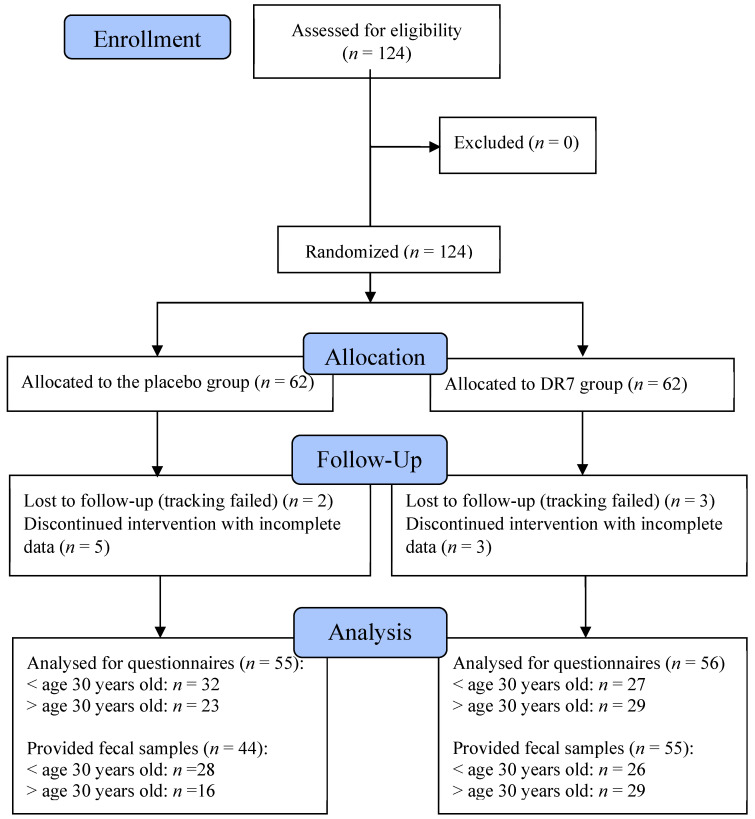
Consort flow chart of recruitment for both intervention groups.

As a continuation of a previous study, the general characteristics for all subjects were as previously reported, where the subjects from both groups fulfilled the inclusion criteria of moderately stressed, and insignificant differences were observed in most of the general and demographic characteristics between placebo and DR7 groups [9]. A total of 124 subjects were assessed for eligibility, recruited, and randomized into either the placebo or DR7 group (Figure 1). A total of 13 subjects dropped-out during the 12-weeks period, either due to failure to track or did not comply in answering the gut health questionnaire, yielding a total of 111 subjects (*n* = 55 for placebo and *n* = 56 for DR7). No adverse effects were reported. A total of 44 subjects from the placebo group and 55 from the DR7 group provided complete sets of faecal samples, yielding a total of 99 completed faecal sample sets.

### 2.2. Gastrointestinal Clinical Outcome

**Figure 2:**
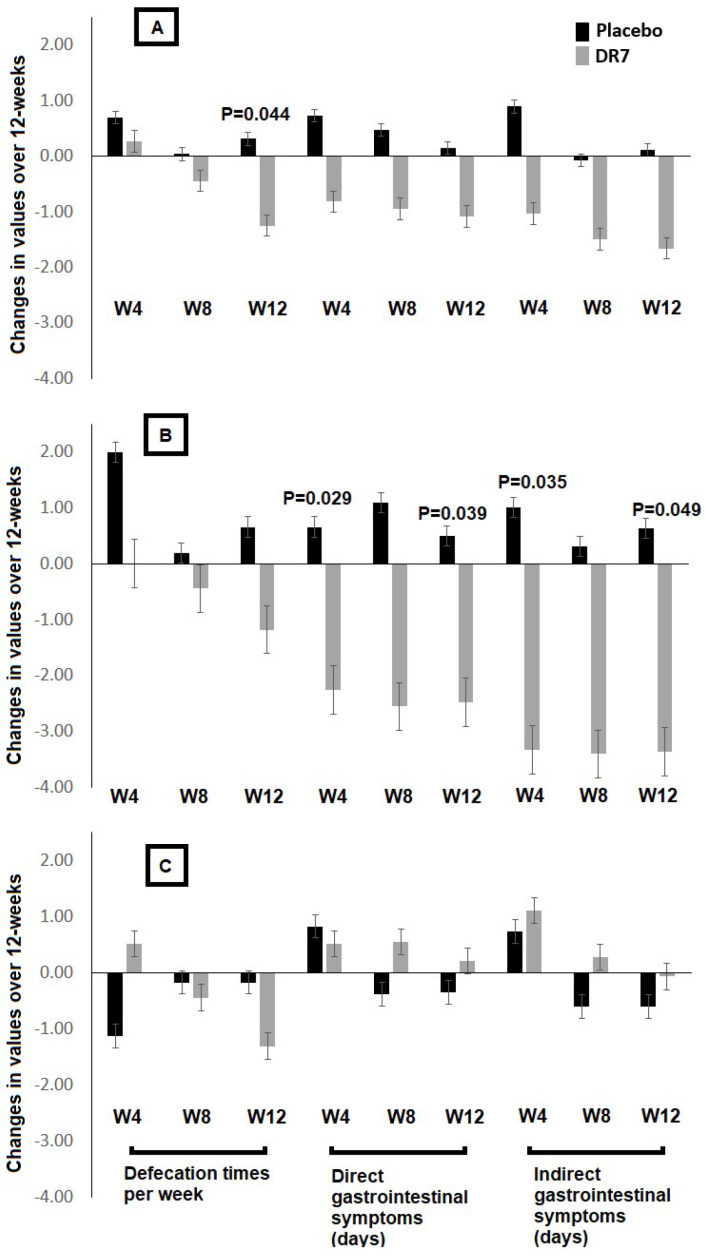
Changes in gastrointestinal clinical outcomes after 12 week administration of *Lactobacillus plantarum* DR7 compared to the placebo group in (**A**) all subjects, (**B**) young adults (aged <30 years old), and (**C**) normal adults (aged >30 years old). Direct gastrointestinal symptoms included vomiting, dysentery (blood in stool), abdominal pain, nausea, and rectal pain (sharp dull, burning, feels like a hard object in the rectum); indirect gastrointestinal symptoms included loss of appetite, fatigue, dizziness, headache, dehydration, and fever; number of defecation times were calculated based on a weekly basis. *p*-values indicated the difference between treatment groups at individual time points. Results are expressed as mean, error bars (SEM); *n* = 111 (DR7 *n* = 56 and placebo *n* = 55).

The gastrointestinal questionnaire was developed to evaluate on several parameters involving defecation frequency and direct and indirect gastrointestinal symptoms, which is relevant for the use in this study. It has been reported that natural disasters such as flood and monsoon often affect gastrointestinal health, attributed to changes in diets, lifestyles, availability of clean water supply, and higher spread of infectious diseases in shelters and evacuation centres [11]. Although monsoons and coastal storms often increase the incidences of diarrhoea attributed to poorer sanitation and contaminated water, infectious diarrhoea has also been reported to rise after floods [12]. The incidences of diarrhoea have been reported by subjects in both placebo and DR7 groups in the present study, although an insignificant difference in frequency was observed between groups over 12 weeks (data not shown). Amid this, the administration of DR7 decreased the frequency of defecation in all subjects as compared to the placebo after week-12 (Figure 2A). This may be explained via the modulatory effects of DR7 against increased bowel movement and defecation as triggered by the central nervous system (CNS) upon stress. Stress affects the regulatory mechanisms and responses of the CNS leading to stimulated colonic motor activity, increased movements of the bowel, and subsequently, increased frequency of defecation [13]. Thus, although DR7 did not exert a significant diarrhoea-reducing effect as compared to the placebo, the administration of DR7 may have alleviated unnecessary bowel movement as induced by stress.

We have previously reported that the administration of DR7 was more effective in reducing symptoms of stress and plasma levels of cortisol in younger adults as compared to the placebo while such an effect was less observed in normal adults [9]. Thus, in the present assessment, we have also evaluated gastrointestinal clinical symptoms in both subgroups. Although the administration of DR7 did not show any significant effects against gastrointestinal disorders in normal adults aged above 30 years old, younger adults below 30 years old seemed to benefit from the administration of DR7, where decreased durations for both direct and indirect gastrointestinal symptoms were observed as compared to the placebo group as early as week-4 and continuously till week-12 (Figure 2B,C). The response to either pain or discomfort-related stressors often increases cortisol secretion leading to a sensitized physiologic stress response. To facilitate the consolidation of fear for survival and avoidance of danger, glucose reserves are mobilized for energy and modulating inflammation by cortisol [14]. Reductions in direct and indirect gastrointestinal symptoms have led to reduced levels of discomfort and subsequently reduced levels of stress and cortisol in younger adults.

### 2.3. Alpha/Beta Diversity

**Figure 3:**
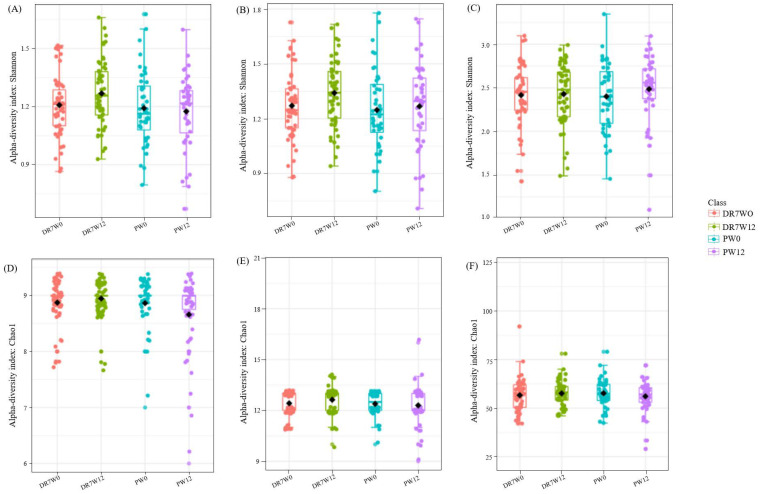
Alpha diversity plots for stressed adults at baseline (W0) and after week-12 (W12), upon administration of *Lactobacillus plantarum* DR7 or placebo. Diversity measured by Shannon Evenness Index for (**A**) class (W0: *p* = 0.264; W12: *p* = 0.034), (**B**) order (W0: *p* = 0.316; W12: *p* = 0.116), and (**C**) genus (W0: *p* = 0.919; W12: *p* = 0.351), and Chao1 Richness Index for (**D**) class (W0: *p* = 0.876; W12: *p* = 0.005), (**E**) order (W0: *p* = 0.781; W12: *p* = 0.018), and (**F**) genus (W0: *p* = 0.416; W12: *p* = 0.123). The line inside the box represents the median, whereas the whiskers represent the lowest and highest values within the interquartile range. Outliers, as well as individual sample values, are shown as dots. Statistical significance was analyzed using the Mann–Whitney *U* test. *n* = 99 (DR7 *n* = 55 and placebo *n* = 44).

**Figure 4:**
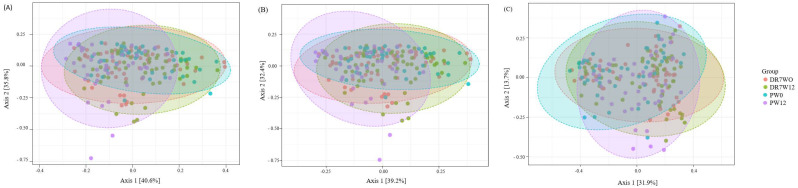
Beta diversity measured by Bray–Curtis dissimilarity and Principal Coordinates Analysis (PCoA) for class (**A**), order (**B**), and genus (**C**) are plotted for stressed adults at baseline (W0) and after week-12 (W12), upon administration of *Lactobacillus plantarum* DR7 or placebo. Diversity in microbial community composition was achieved by permutational analysis of variance (PERMANOVA) for class, order, and genus. PERMANOVA: class (W0: *p* = 0.343, W12: *p* < 0.001), order (W0: *p* = 0.338, W12: *p* < 0.001), and genus (W0: *p* = 0.338, W12: *p* < 0.001). *n* = 99 (DR7 *n* = 55 and placebo *n* = 44).

Alpha diversity measures differences within samples. The Chao1 Index provides a measure of alpha diversity in terms of operational taxonomic unit (OUT) “richness,” equally taking into account frequent and rare OTUs. Meanwhile, the Shannon Diversity Index also considers OTU’s “evenness,” as it takes into account the frequency of each OTU [15]. Both study groups were comparable in terms of richness and evenness at baseline at the class, order, and genus level (Figure 3). However, at the end of the intervention period, the placebo group displayed significantly lower richness compared to DR7 at both class (*p* = 0.005) and order (*p* = 0.018) levels, as well as reduced evenness at class level (*p* = 0.034). Our present data showed that the administration of DR7 prevented the reduction of within-group ecological diversity while taking into account the number of different taxa and relative abundances, which was evidently reduced in the placebo group over time. Beta diversity measures differences between samples. The Bray–Curtis Index considers both the co-occurrence and differential abundance of OTUs. Compositional differences were not observed at week-0 (Figure 4). However, at the end of the intervention, significant differences were consistently observed between DR7 and placebo at class, order, and genus levels (all with *p* < 0.001). These indicated that DR7 prevented a shift in microbial community compositional changes over time, which evidently occurred in the placebo group. A recent medium-scaled study involving 671 human subjects showed that a lower gut microbial diversity was associated with increased levels of stress and anxiety, accompanied by an altered overall composition of the gut microbial community [16]. In addition, gut microbiota diversity has been greatly reported to play an important role in maintaining the stability of the intestinal ecosystem as well as normal ecological functions. A reduced microbial diversity has been reported in an array of gastrointestinal diseases such as Crohn’s disease [17] and inflammatory bowel disease (IBD) [18], while milder cases such as diarrhea have been associated with decreased phylotype richness [19]. Recent evidence has also shown that a shift in gut microbiota diversity is associated with mental health and psychiatric disorders such as stress, sociability, cognition, anxiety, depression, and autism [20]. Young patients with attention deficit hyperactivity disorder were reported to display reduced alpha diversity and differ in microbial composition as compared to healthy controls [15].

Animal studies have shown that stress caused reduced abundance and diversity in gut microbiota profiles, where stressed pregnant monkeys showed lower abundance of lactobacilli and bifidobacterial [21], mice exposed to social disruption stress led to a shifted colonic clustering compared to the control as observed via beta diversity Principal Coordinates Analysis (PCoA) plots accompanied by a reduced abundance of *Porphyromonadaceae* and *Lactobacillaceae* [22], while depressed mice due to chronic mild stress showed a reduced abundance of *Lactobacillus* and *Turicibacter* [23]. A study involving undergraduate students also showed reduced total faecal microbial load amid exam stress [24]. Amid stress, within-sample microbial richness reduced leading to increased intersample dissimilarities in the present study. The administration of DR7 has aided in the maintenance of gut microbial richness and evenness, to prevent a shift in diversity amid stress.

### 2.4. Compositional Changes Between DR7 and Placebo

As alpha and beta diversity analyses yielded significant changes between DR7 and placebo groups over 12 weeks, we further analysed faecal microbiota changes along different taxonomic levels. Compared to DR7, subjects administered placebo had a drop in phylum Bacteroidetes over 12 weeks (Supplementary Figure S2; *p* < 0.001), which could be traced to subsequent taxonomic levels of class Bacteroidia (Supplementary Figure S3; *p* < 0.001) and order Bacteroidales (Supplementary Figure S4; *p* < 0.001). Of note, a lower count of Bacteroidetes has been observed in patients with altered colonic movements such as young children hospitalized for infectious diarrhoea [25] and patients with functional constipation [26].

Conversely, although both study groups displayed a comparable abundance of phylum Firmicutes (and of lower taxonomic levels class Clostridia and order Clostridiales), these bacteria were reduced at the end of the study in subjects receiving DR7, while were increased in the placebo group, resulting in significant differences at phylum (Supplementary Figure S2; *p* = 0.002), class (Supplementary Figure S3; *p* = 0.001), and order Clostridiales (Supplementary Figure S4; *p* = 0.001). These differences could be further traced down to genera *Blautia* and *Romboutsia* (Supplementary Figure S5; *p* < 0.001), which were increased in the placebo group at the end of the study. This effect was compensated by a larger abundance of class Negativicutes (Supplementary Figure S3; *p* = 0.003) and order Selenomonadales (Supplementary Figure S4; *p* = 0.003) in subjects consuming DR7 as compared the placebo group. Of note, although both groups showed a decrease in the abundance of the genus *Acidaminococcus* (belonging to order Selenomonadales), the administration of DR7 contributed to a larger decrease than the placebo over 12 weeks (Supplementary Figure S5; *p* = 0.001). Orders Clostridiales and Selenomonadales are largely gut commensal inhabitants without exerting much detrimental effects on hosts. At the genus level, although *Blautia* has been reported as a butyrate producer that is beneficial for colonocytes, a higher abundance of *Blautia* has also been reported in patients with inflammatory pouchitis [27], nonalcoholic fatty liver disease [28], systemic lupus erythematosus [29], and breast cancer [30]. *Acidaminococcus* has been positively correlated with stunting heights in malnourished children [31], attributed to its fastidious requirement for glutamate as a sole source of carbon and energy, where glutamate in the gut is an essential oxidative fuel for intestinal epithelium [32].

Subjects administered with DR7 had an increased abundance of class Deltaproteobacteria (Figure S3; *p* < 0.001), order Desulfovibrionales (Supplementary Figure S4; *p* < 0.001), and genera *Bilophila* (Supplementary Figure S5; *p* < 0.001) and *Desulfovibrio* (*p* = 0.001) as compared to subjects on placebo, which showed a decreased abundance over 12 weeks. Although many classes of the phylum Proteobacteria are associated with human pathogens such as Alphaproteobacteria (for the genera *Brucella* and *Rickettsia*), Betaproteobacteria (for the genera *Bordetella* and *Neisseria*), Gammaproteobacteria (for the genera *Escherichia*, *Shigella*, *Salmonella*, and *Yersinia*), and Epsilobacteria (for the genus *Helicobacter*), genera *Bilophila* and *Desulfovibrio* of the class Deltaproteobacteria are widely identified as sulphate reducers yielding hydrogen sulphide (H_2_S) in the gut. Although past knowledge has associated H_2_S with damages of the gut and onset of IBD, recent advances have shown that H_2_S is an important mediator for gastrointestinal mucosal defence, repairing of epithelial injury, preventing dysbiosis due to the use of nonsteroidal anti-inflammatory drugs, and promoting resolution of inflammation [33]. The increased abundance of levels along the lineage of Deltaproteobacteria seemed compensated by a decreased abundance in subjects consuming DR7 for the class of Actinobacteria (Supplementary Figure S3; *p* = 0.011), order Actinomycetales (Supplementary Figure S4; *p* = 0.002), and genus *Actinomyces* (Supplementary Figure S5; *p* < 0.001) as compared to subjects on placebo over 12 weeks. Although Actinobacteria is commensal for the human oral mucosa, nasopharyngeal, gastrointestinal, and urogenital tracts, the *Actinomyces* genus has been isolated from colon, cecum, and appendix of patients with actinomycosis, primarily attributed to its ability to form biofilm, induce inflammation, and aggravate injuries caused by inflammation [34].

In addition to physiological and pathophysiological processes, stress has also been reported to affect the gut environment, namely, loss of gut barrier functions [35], relapses of IBD [36], and structural weakening of the colonic mucosal layers [37]. DR7 had modulated gut microbiota as a first targeted site, leading to a healthier gut ecosystem that subsequently alleviated the stress clinical outcomes and inflammatory parameters as observed in our previous study [9].

**Figure 5:**
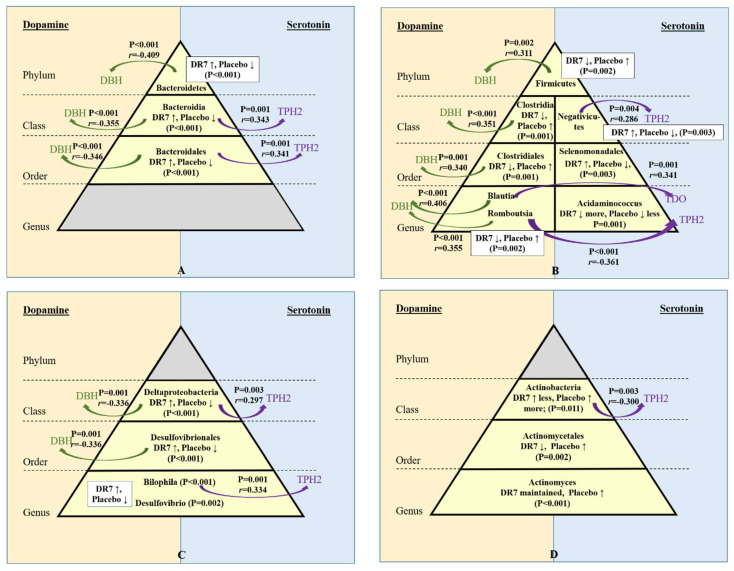
Changes in gut microbiota after 12-week administration of *Lactobacillus plantarum* DR7 compared to placebo group along the lineage of phylum Bacteroidetes (**A**) and Firmicutes (**B**) and classes Deltaproteobacteria (**C**) and Actinobacteria (**D**). Only microbiota groups which showed significant changes over 12 weeks between placebo and DR7 groups are shown (as indicated by *p*-values inside the pyramid; Mann–Whitney *U* test; ↑ indicates an increase over 12 week and ↓ indicates a reduction over 12 weeks). Curved arrows indicate correlations between gut microbiota and blood gene expressions of dopamine *β*-hydroxylase (DBH), tryptophan hydroxylase-2 (TPH2), and tryptophan 2,3-dioxygenase (TDO) (*p*-value and rho (*r*) obtained from Spearman’s rank correlations. *n* = 99 for gut microbiota (DR7 *n* = 55 and placebo *n* = 44); *n* = 111 for blood gene expressions (DR7 *n* = 56 and placebo *n* = 55).

Limitations have been raised on the accuracy and use of higher taxonomic levels such as phylum to predict and correlate with diseases and biological functions, attributed to the vast diversity along lower taxonomic levels of the human gut microbiota. It is thus crucial to note that in our present study, the changes in gut microbiota upon the administration of DR7 was consistent along different lower taxonomic levels such as that along the phyla of Bacteroides and Firmicutes (Figure 5A,B), and those along the classes of Deltaproteobacteria and Actinobacteria (Figure 5C,D).

### 2.5. Correlation of Gut Microbiota and Stress Neurotransmitters

We have previously reported that DR7 reduced plasma cortisol levels and exerted changes along the pathways of two neurotransmitters, namely, serotonin, and dopamine. The administration of DR7 for 12-weeks had lowered the expressions of dopamine β-hydroxylase (DBH) and tyrosine hydroxylase (TH) along the dopamine pathway and also lowered the expressions of indoleamine 2,3-dioxygenase (IDO) and tryptophan 2,3-dioxygenase (TDO), while increasing the expressions of tryptophan hydroxylase-2 (TPH2) and 5-hydroxytryptamine receptor-6 (5-HT6) along the serotonin pathway as compared to the placebo [9]. Thus, in the present study, correlation analyses were performed to evaluate correlations between microbiota groups that were changed upon the administration of DR7, with those of genes involved along the serotonin and dopamine pathways. Although plasma cortisol levels and defecation frequency did not show any significant correlations with gut microbiota profiles, our present correlation analyses showed significant correlations involving DBH, TPH2, and TDO (Figure 5). We have previously reported that upon the administration of DR7, the expressions of TPH2 in blood was higher than that of placebo by 3.7 times, while the expressions of DBH and TDO was lower by 1.1 times and 1.3 times than the placebo, respectively [9]. Here, we report these changes correlated to changes in the relative abundance of specific bacterial groups in the gut microbiota. It is noted that baseline TPH2 expression in blood was low, as TPH2 is primarily expressed in the brain, whereas TPH1 is expressed in the brain, gastrointestinal tract, and pituitary glands [38]. However, no significant correlation was observed for TPH1.

Although changes in gut microbiota upon the administration of DR7 was consistent along different taxonomic levels, the correlations of the changes in these microbial groups with changes in neurotransmitters gene expression also showed such consistency. The phylum Bacteroidetes showed a negative correlation with gene expression of DBH (*p* < 0.001, *r* = −0.409; Figure 5A), which was consistent along lower taxonomic levels such as class Bacteroidia (*p* < 0.001, *r* = −0.355) and order Bacteroidales (*p* < 0.001, *r* = −0.346). DBH catalyses the conversion of dopamine to norepinephrine, an indication of increased stress as seen in the placebo group with a lower abundance of Bacteroidetes, Bacteroidia, and Bacteroidales. As the levels of cortisol increase, the brain noradrenergic is activated, where the postsynaptic effects of norepinephrine are triggered to induce alertness, awareness, wakefulness, and also attention amid stressful conditions. Bacteroidia and Bacteroidales, which were maintained upon the administration of DR7 compared to placebo also showed positive correlations with TPH2 (*p* = 0.001; Figure 5A). TPH2 converts tryptophan to serotonin in the brain, where an imbalanced level of serotonin has been reported in patients with psychological disorders including mood and anxiety [39]. Bacteroidetes are reportedly reduced in children with autism [40] and patients with dementia [41], whereas patients with depression have been reported to have lower abundance of Bacteroidia and Bacteroidales [42].

A positive correlation was observed between gene expression of DBH and taxonomic levels along the phylum Firmicutes (*p* = 0.002, *r* = 0.311), class Clostridia (*p* < 0.001, *r* = 0.351), order Clostridiales (*p* = 0.001, *r* = 0.340), and genera *Blautia* (*p* < 0.001, *r* = 0.406) and *Romboutsia* (*p* < 0.001, *r* = 0.355) (Figure 5B). The administration of DR7 had also prevented the increase in abundance of all taxonomic levels along the phylum Firmicutes, whereas the placebo showed an increase amid stress. *Blautia* also has a positive correlation with TDO (*p* = 0.001, *r* = 0.341), which competes with TPH2 for tryptophan in the brain for the conversion into kynurenine. Reduced level of serotonin and increased level of kynurenine has been shown in patients with anxiety and depressive disorders [39]. *Romboutsia* has a negative correlation with TPH2 (*p* < 0.001, *r* = −0.361), whereas class Negativicutes, which was increased in abundance upon the administration of DR7, showed a positive correlation with TPH2 (*p* = 0.004, *r* = 0.286). Although DR7 prevented the increase in abundance of all taxonomic levels along the phylum Firmicutes, several other studies have associated Firmicutes with mental health, where a higher abundance of Firmicutes was seen in demented patients compared to nondemented controls [41], Clostridiales was more abundant in patients with major depression [43], and a higher abundance of *Blautia* was observed in patients with Alzheimer’s Disease [37] and major depression disorder [44].

Deltaproteobacteria and its lower taxonomic level of order Desulfovibrionales which were both increased in abundance upon administration of DR7 showed negative correlations with DBH (*p* = 0.001, *r* = −0.336; Figure 5C). Deltaproteobacteria also showed a positive correlation with TPH2 (*p* = 0.003, *r* = 0.297), while its genus of *Bilophila* also showed a similar trait (*p* = 0.001, *r* = 0.002). Meanwhile, Actinobacteria, which showed a lesser increase in abundance upon administration of DR7 as compared to the placebo, exhibited a negative correlation with TPH2 (*p* = 0.003, *r* = −0.300; Figure 5D). Past reports have shown different associations of these microbial groups with different mental disorders. Bilophila was shown to decrease in abundance, in subjects with autism spectrum disorders [45] but increased in abundance in patients with Alzheimer’s Disease [37]. Although Actinobacteria has been positively associated with signalling in the thalamus, hypothalamus, and amygdala leading to better cognitive speed, attention, and flexibility in humans [46], 16s rRNA sequences of Actinobacteria were found more abundant in frozen and fixed autopsied brain samples from patients with multiple sclerosis [47].

While serotonin is one of the main brain neurotransmitters, approximately 90% of total serotonin in humans is located in enterochromaffin cells in the gastrointestinal (GI) tract [48], where serotonin plays important roles in promoting immunity and reducing inflammation in various models of mucosal infections [49]. This is in contrast with the other metabolite of tryptophan and kynurenine, where an increased conversion to kynurenine from tryptophan has been reported to increase incidences of upper respiratory tract infections (URTI) [50]. We have previously reported the effects of DR7 on improving symptoms of URTI via modulating systemic immunity and inflammatory responses in adults [51]. Information on the effects of gut microbiota on the pathways of dopamine remains limited as compared to that of serotonin, where a decrease in serotonin level has been associated with the absence of certain gut bacteria in germ-free animals as early as 1967 [52]. As serotonin is produced in gut by enterochromaffin cells while gut microbiota influences the number and function of enterochromaffin cells thereby promoting the release of serotonin in the gut, serotonin has been widely evaluated for gut–brain axis properties. Meanwhile, dopamine and norepinephrine are reportedly produced by gut microbiota thus associated to play some roles as signalling molecules mediating the function of the microbiota–gut–brain axis less than 10 years ago [53]. Emerging studies in all these years have thus reported on the effects of gut microbiota on serotonin and dopamine levels, and in certain general extents, reported on some psychological effects. To our best knowledge, the present data is the first to report a correlation between gut microbiota and genes related to the serotonin and dopamine pathways, which is also consistent across multiple taxonomic levels.

## 3. Materials and Methods

### 3.1. Lactobacillus plantarum DR7 and Placebo Products

*Lactobacillus plantarum* DR7 was isolated from fresh cow’s milk in Penang, Malaysia [9]. The preserved stock cultures of DR7, in 20% glycerol at −20 °C, were activated in sterile De Man, Rogosa, Sharpe (MRS) broth (Hi-media, Mumbai, India) for three successive times using 10% (*v*/*v*) inoculums and incubated at 37 °C for 24 h [54]. The identity of all working cultures was reconfirmed using 16S rRNA sequencing and two strain-specific primers of (i) (F) GCAAGGCCACTTGATCGTTG and (R) AATCAGTCGCATCCAGCCAA and (ii) (F) AGCCATTCTCAGTTCGGATTGT and (R) GCTCTTGTTCGACTTCCCCTAA. PCR amplification of 16S rDNA was performed using the universal 16S rDNA primer, with the forward sequence, 27F; 5′-AGAGTTTGATCCTGGCTCAG-3′ and reverse primer sequence, 1492R; 5′-GGTTACCTTGTTACGACTT-3′, in a thermal cycler (Bio-Rad, Hercules, CA, USA). The program for amplification consisted of (i) denaturation at 95 °C for 5 min, (ii) 35 cycles of denaturation at 95 °C for 30 s, annealing at 52 °C for 1 min, and extension at 72 °C for 2 min, and (iii) final extension at 72 °C for 4 min, and finally, maintained at 4 °C until further use. The PCR amplicons were subjected to agarose gel electrophoresis at 100 V for 60 min and visualized using the GeneGenius Imaging System (Syngene, Cambridge, UK). The PCR products purification and sequencing were performed by Apical Scientific Sdn Bhd. The nucleotide sequences of the isolates were analysed using the BLAST program from NCBI (http://www.ncbi.nlm.nih.gov). The accession number for the whole genome of DR7 is CP031318. Both DR7 and placebo products were manufactured by GN Pharmaceuticals Sdn. Bhd., Selangor, Malaysia under GMP-certified manufacturing plant which was also certified HALAL by JAKIM, Malaysia. Live cultures of DR7 is proven stable in a lactose-free medium such as soymilk at 4 °C for 168 h (Supplementary Figure S1). Daily consumption consisted of one aluminium sachet (2 g) containing light-yellow powder of either 10^9^ cfu/sachet of DR7 and maltodextrin as excipient or placebo with only maltodextrin. All products were stored away from direct sunlight and at below 30 °C.

### 3.2. Selection of Subjects

Subjects were recruited from Penang and Kubang Kerian, Malaysia, and screened based on the inclusion and exclusion criteria. The inclusion criteria were men or women, aged 18–60 years old, willing to commit throughout the experiment, and scored moderate stress level on Cohen’s Perceived Stress Scale (PSS-10) [55]. The exclusion criteria included subjects with type-I diabetes, taking term medication due to certain severe illness, having HIV/AIDS, deficient in glucose-6-phosphate dehydrogenase, and who, in the opinion of the investigator, were not likely to complete the trial for whatever reasons. Before the start of the study, written informed consent was obtained from all participating subjects.

### 3.3. Study Protocol

This was a double-blind, randomized, and placebo-controlled design study. Qualified subjects were randomized according to a 1:1 ratio to the two arms of the study according to a computer-generated list with treatment codes. This list was prepared by the study statistician, who had no contact with the participants, and the allocation sequence was not available to any member of the research team until the end of the study. This study was conducted according to the guidelines laid down in the Declaration of Helsinki. All procedures involving human subjects were approved by the JEPeM-USM Review Panel on Clinical Studies (Approval number USM/JEPeM/17040228, 24 August 2017) and was registered at ClinicalTrials.gov (identifier number NCT 03370458, 12 December 2017). The sample size was calculated for a parallel-group study design involving one prevention arm and one placebo arm and was based on power design analysis as previously described [9], where a total of 124 subjects were needed comprising 62 subjects in each group (treatment and placebo; inclusive of 15% dropout).

### 3.4. Analyses

#### Questionnaires

Eligible subjects were requested to complete a gut health condition questionnaire at baseline, week-4, week-8, and week-12, which recorded the occurrence for direct gastrointestinal symptoms (such as vomiting, dysentery (blood in stool), abdominal pain, nausea, rectal pain (sharp dull, burning, and feels like a hard object in the rectum)), indirect gastrointestinal symptoms (such as loss of appetite, fatigue, dizziness, headache, dehydration, and fever), and the number of defecation times. The development of all questionnaires as assessment tools also included translational processes to the Malay and Chinese languages, where all versions were validated via stages of development and face validation [56,57] and were also used in our previous study on gastrointestinal health [58].

### 3.5. Gut Microbiota Analyses

#### 3.5.1. DNA Extraction, PCR Amplification, and Sequencing

Faecal samples were collected at baseline (week-0) and week-12 in faecal collection tubes containing RNAlater™ solution (Qiagen, Hilden, Germany) and glass beads by the subjects and stored at refrigerated temperature (4–8 °C) for not more than 3 days, prior to delivery to the laboratory and stored at −80 °C until further analyses. The faecal samples were homogenized by vortexing the tube containing glass beads prior to DNA extraction and purification as previously described [10,11]. Purified DNAs were determined by NanoDrop 2000 UV-Vis Spectrophotometer (Thermo Scientific, Wilmington, NC, USA). The V3–V4 hypervariable regions of the bacteria 16S rRNA gene were amplified with primers 341F (5′-CCTAYGGGRBGCASCAG-3′) and 806R (5′-GGACTACHVGGGTWTCTAAT-3′) by thermocycler PCR system (GeneAmp 9700, ABI, San Diego, CA, USA). The PCR reactions were performed in triplicates with 20 μL mixture containing 4 μL of 5 × FastPfu Buffer, 2 μL of 2.5 mM dNTPs, 0.8 μL of each primer (5 μM), 0.4 μL of FastPfu Polymerase, and 10 ng of template DNA, in the following sequence: 3 min of denaturation at 95 °C, 27 cycles of 30 s at 95 °C, 30 s for annealing at 55 °C, and 45 s for elongation at 72 °C, and a final extension at 72 °C for 10 min. The PCR products were then extracted from a 2% agarose gel and further purified using the AxyPrep DNA Gel Extraction Kit (Axygen Biosciences, Union City, CA, USA) and quantified using QuantiFluor™-ST (Promega, Madison, WI, USA) according to the manufacturer’s protocol. The purified amplicons were pooled in equimolar and paired-end sequenced (2 × 300) on an Illumina MiSeq platform (Illumina, San Diego, CA, USA).

#### 3.5.2. Bioinformatics Analysis on 16s rRNA Gene Profiling

The 16s rRNA gene sequences were processed using QIIME v.1.9.1 (ref QIIME allows analysis of high-throughput community sequencing data) and USEARCH v.10.0 (ref Search and clustering orders of magnitude faster than BLAST). Raw FASTQ files were quality filtered by Trimmomatic and merged by USEARCH with the following criteria: removing of barcodes and primers, filtering of low-quality reads, and finding nonredundancy reads. The merged raw reads were at least 50,000 per sample. Operational taxonomic units (OTUs) were clustered with 97% similarity cut-off using UPARSE. The taxonomy for each 16S rRNA gene sequence was analysed by the RDP Classifier algorithm (http://rdp.cme.msu.edu/) against the Silva 132 16S rRNA database using 60% confidence threshold.

Alpha (within-sample richness) and beta-diversity (between-sample dissimilarity) estimates were computed using MicrobiomeAnalyst phyloseq-R package version 3.6.1 (https://www.microbiomeanalyst.ca/MicrobiomeAnalyst/home.xhtml) for class, order and genus.

### 3.6. Statistical Analyses

Data were analysed using SPSS version 20.0 (SPSS Inc, Chicago, IL, USA). The primary hypothesis of this study involved differential efficacy between the two treatment groups of DR7 and placebo. Considering the skewed distribution and nonparametric nature of our data, differences in OTU relative abundance between DR7 and placebo groups were compared using the Mann–Whitney *U* test, whereas the correlations between OTUs relative abundance and gene expression data were evaluated using Spearman’s rank correlations with rho (*r*) as the correlation coefficient. Alpha diversity of gut microbiota was measured by Shannon and Chao1 Indexes and compared using Mann–Whitney *U* test, whereas beta diversity was calculated by principal coordinates analysis (PCoA) on Bray–Curtis dissimilarity and compared using permutational analysis of variance (PERMANOVA).

Data of blood gene expressions of dopamine *β*-hydroxylase (DBH), tryptophan hydroxylase-2 (TPH2), and tryptophan 2,3-dioxygenase (TDO) used in correlation analyses were obtained from our previous study [9]. All tests were two-sided with *p* < 0.05 as considered statistically significant. Both differences in relative abundance and correlation analyses were adjusted for multiplicity based on the number of OTUs detected at each taxonomical level (class, order, and genus) by using Benjamini and Hochberg procedure at a False Discovery Rate (FDR) threshold of 0.1.

## 4. Conclusions

Taken altogether, our present data showed that the administration of DR7 modulated stress-induced bowel movement by decreasing the frequency of defecation as compared to the placebo after 12 weeks. Alpha diversity analysis also showed that DR7 prevented the decrease of gut microbiota OTU’s richness and evenness, whereas beta diversity analysis showed that DR7 maintained the distribution of gut microbial profiles amid stress. It is noted that these changes in gut microbiota upon the administration of DR7 was consistent along different taxonomic levels along the phyla of Bacteroides and Firmicutes and the classes of Deltaproteobacteria and Actinobacteria. Correlation analyses subsequently revealed that the changes of gut microbiota along different taxonomic levels were consistent with several gene expressions of key enzymes involved along the neurotransmitter pathways of serotonin and dopamine. To our knowledge, this is the first study to correlate the effects of a probiotic towards gut microbiota changes and brain neurotransmitters genes, strengthening the hypothesis of health benefits along the gut–brain axis. Considering that probiotic microorganisms are frequently utilized in foods, the current finding will benefit the food industries by enabling the development of new functional food products specifically targeting those for mental health promotions.

## Supplementary Materials

Supplementary materials can be found at https://www.mdpi.com/1422-0067/21/13/4608/s1.

## Abbreviations

CNS	Central nervous system
DBH	Dopamine β-hydroxylase
GI	Gastrointestinal
MDD	Major depressive disorders
MRS	De Man, Rogosa, Sharpe
OTUs	Operational taxonomic units
PCoA	Principal coordinates analysis
PERMANOVA	Permutational analysis of variance
URTI	Upper respiratory tract infections
TDO	Tryptophan 2,3-dioxygenase
TPH2	Tryptophan hydroxylase-2
