# Supplementary material for: Lactobacillus plantarum DR7 Modulated Bowel Movement and Gut Microbiota Associated with Dopamine and Serotonin Pathways in Stressed Adults
Source: Int J Mol Sci. 2020 Jun 29;21(13):4608. doi: 10.3390/ijms21134608 (PMC7370301; doi:10.3390/ijms21134608)
Supplement: Supplementary file 1 [file ijms-21-04608-s001.zip › Supplementary Material.docx]

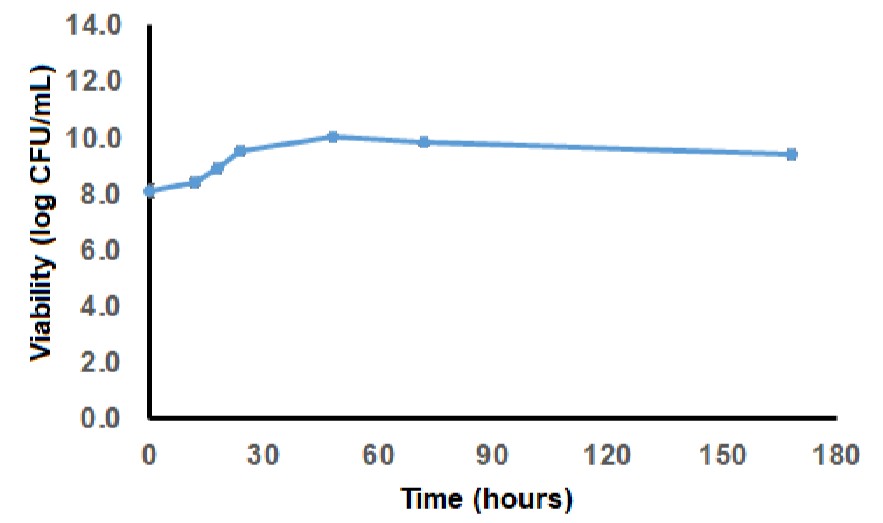


Supplementary Figure 1: Stability of *Lactobacillus plantarum* DR7 in soymilk over 168 hours at 4 °C. Viability maintained above 10^8^ cfu/ml. Results are expressed as mean; error bars (SEM). N=3.


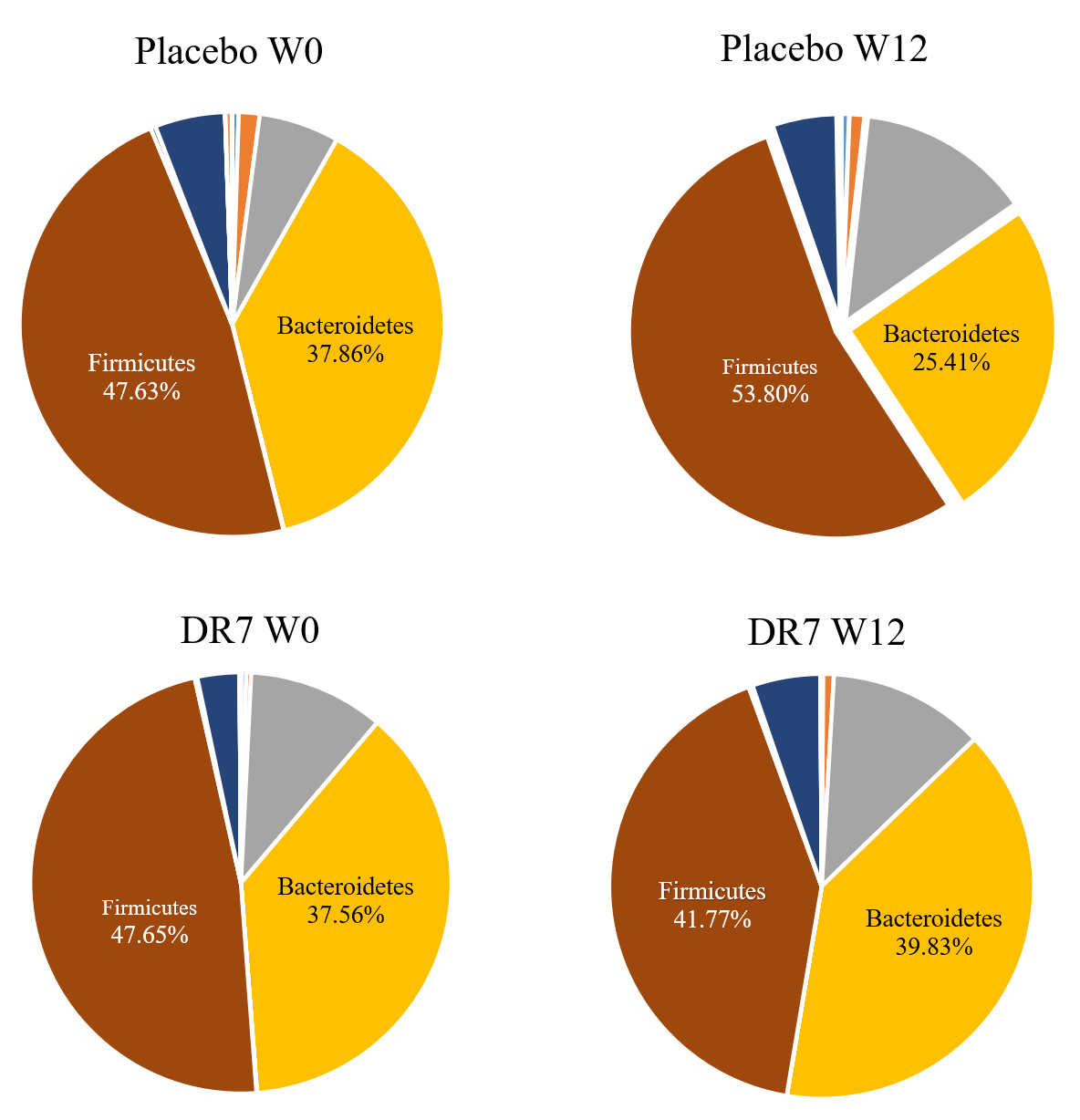


Supplementary Figure 2: Relative abundance of phyla analyzed from fecal samples of stressed adults at baseline (week-0) and after week-12, upon administration of *Lactobacillus plantarum* DR7 or placebo; n=99 (DR7 n=55, placebo n=44).


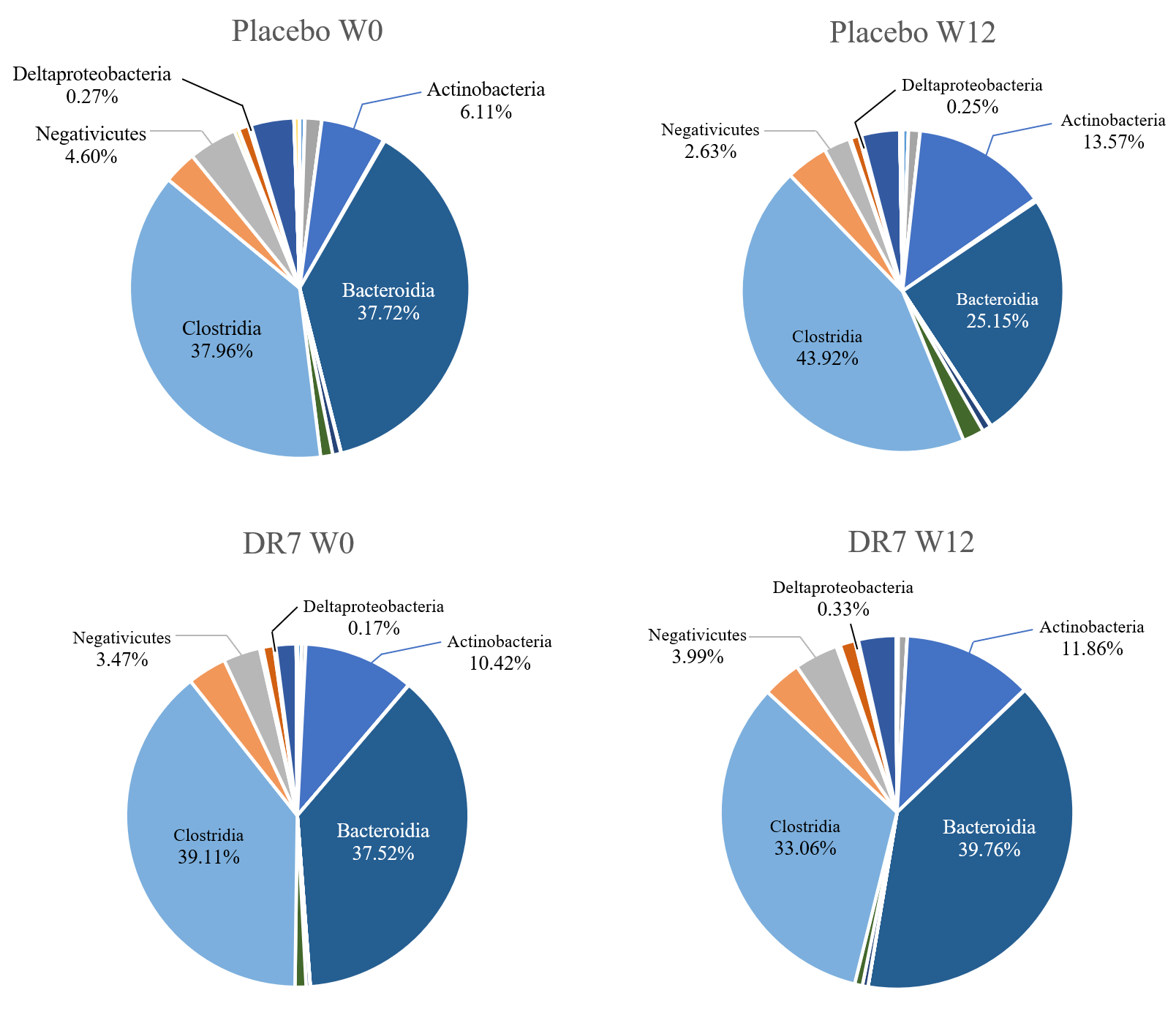


Supplementary Figure 3: Relative abundance of classes analyzed from fecal samples of stressed adults at baseline (week-0) and after week-12, upon administration of *Lactobacillus plantarum* DR7 or placebo; n=99 (DR7 n=55, placebo n=44).


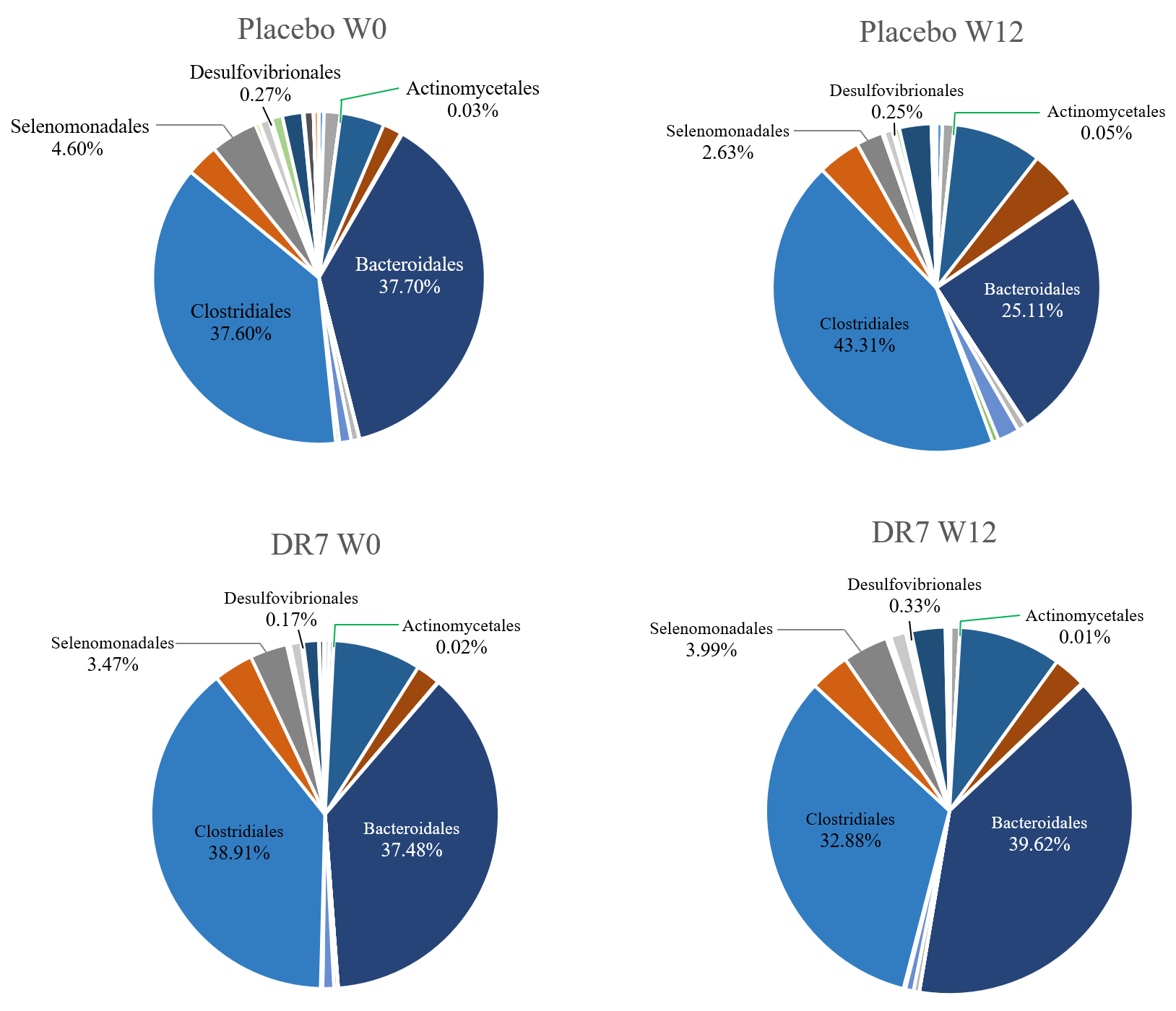


Supplementary Figure 4: Relative abundance of orders analyzed from fecal samples of stressed adults at baseline (week-0) and after week-12, upon administration of *Lactobacillus plantarum* DR7 or placebo; n=99 (DR7 n=55, placebo n=44).


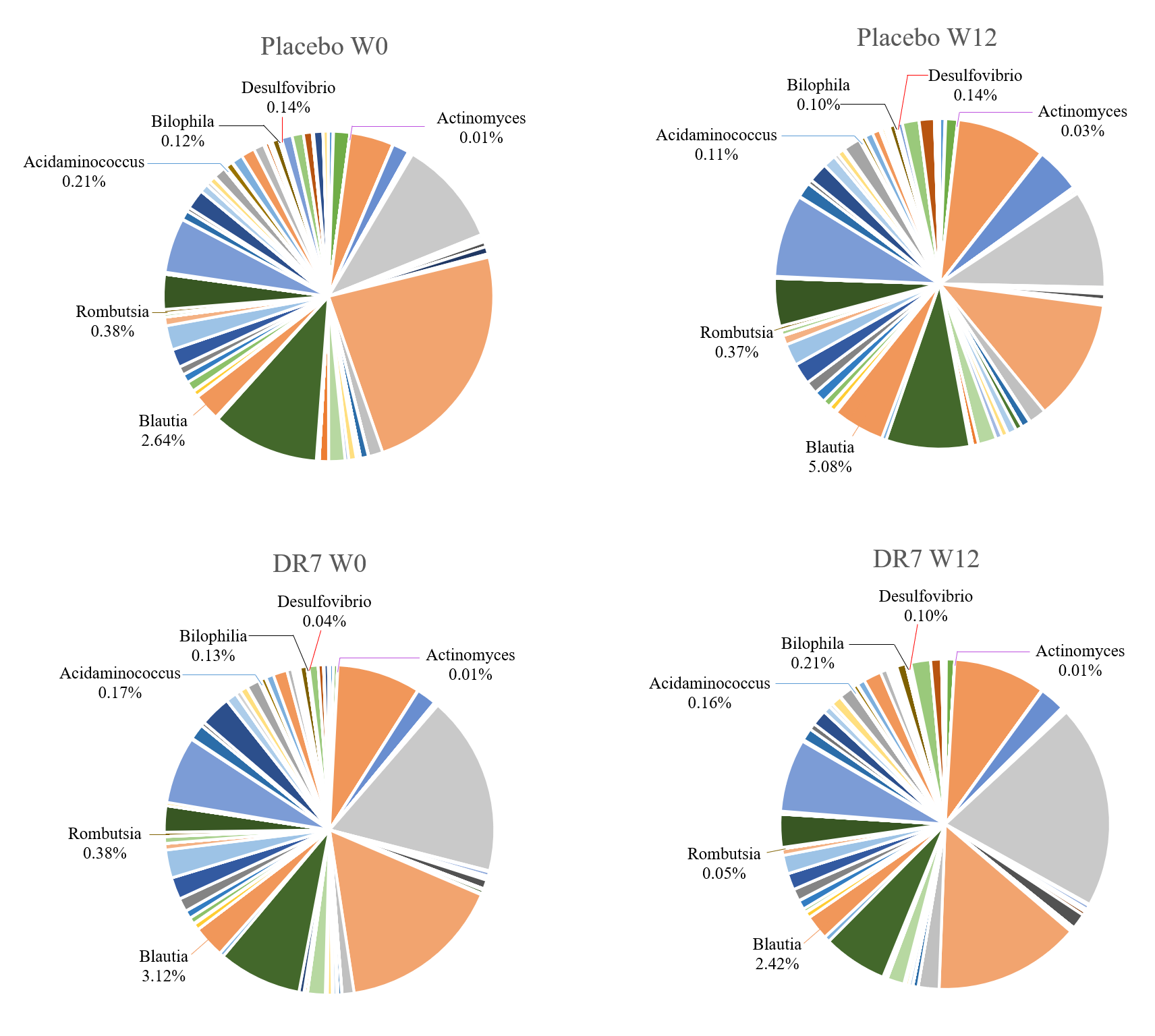


Supplementary Figure 5: Relative abundance of genera analyzed from fecal samples of stressed adults at baseline (week-0) and after week-12, upon administration of *Lactobacillus plantarum* DR7 or placebo; n=99 (DR7 n=55, placebo n=44).
