# Supplementary figures and images for: Lactobacillus plantarum DR7 Modulated Bowel Movement and Gut Microbiota Associated with Dopamine and Serotonin Pathways in Stressed Adults
Source: Int J Mol Sci. 2020 Jun 29;21(13):4608. doi: 10.3390/ijms21134608 (PMC7370301; doi:10.3390/ijms21134608)

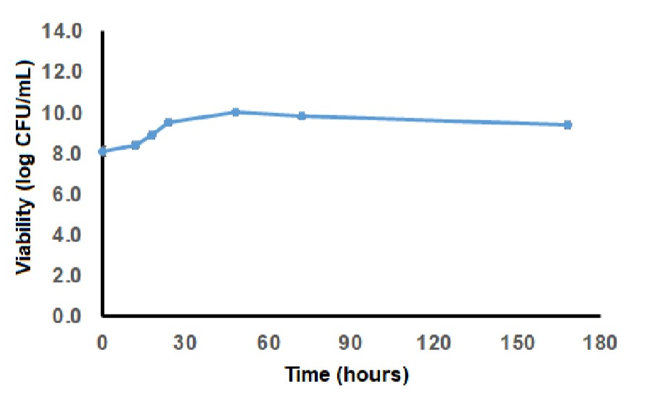

Supplement: Supplementary file 1 [file ijms-21-04608-s001.zip › Figure S1.tif]

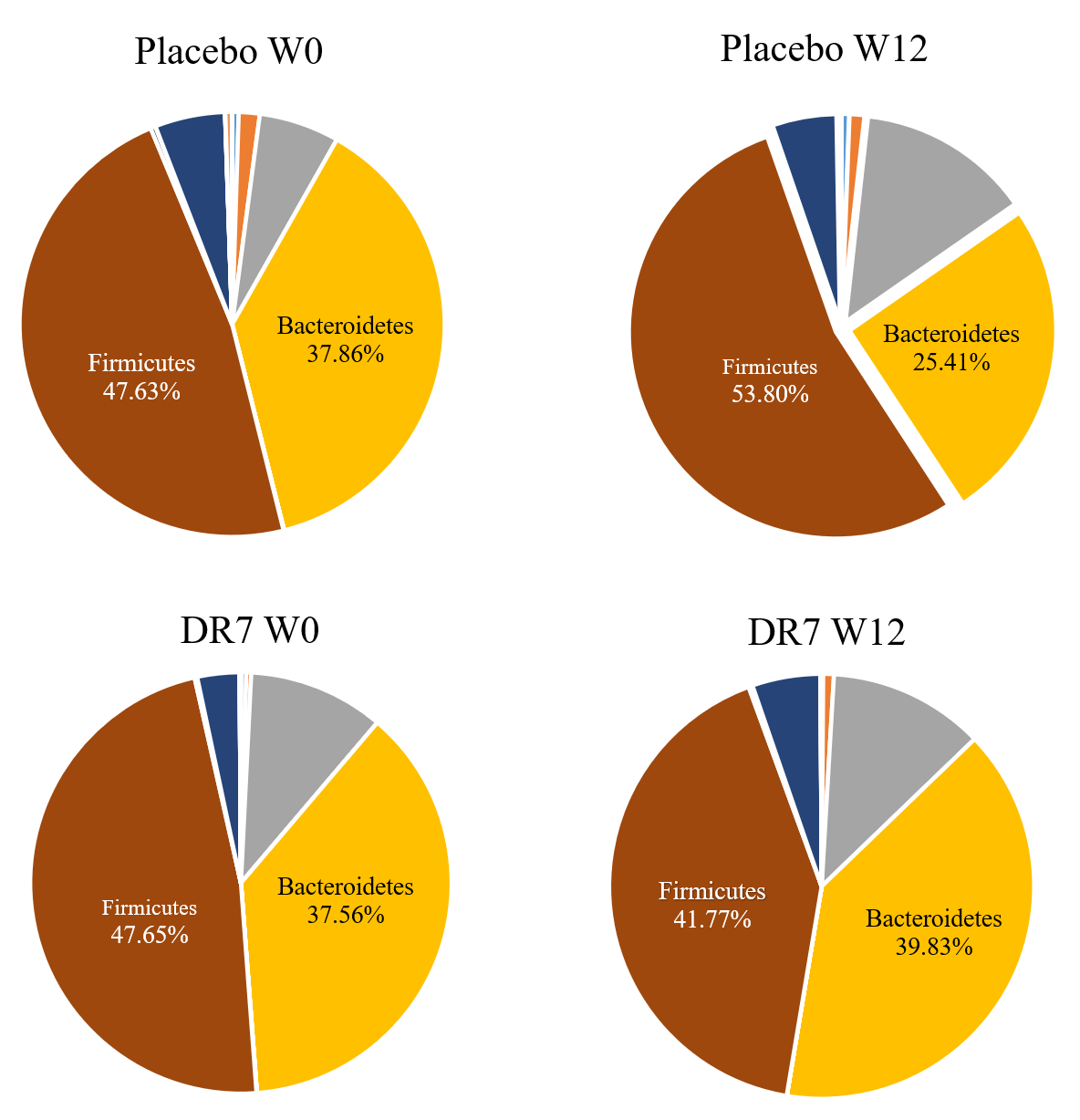

Supplement: Supplementary file 1 [file ijms-21-04608-s001.zip › Figure S2.tif]

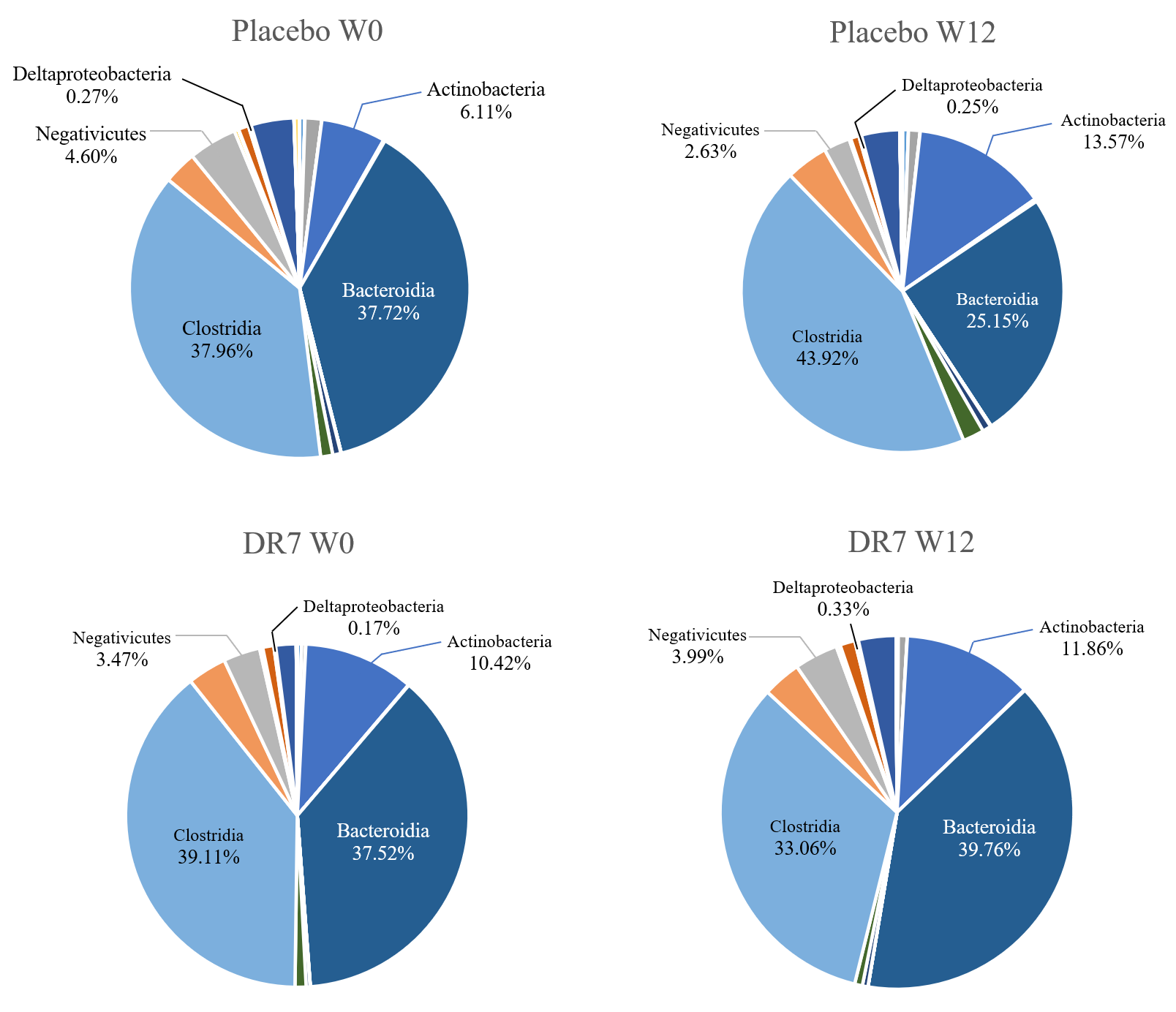

Supplement: Supplementary file 1 [file ijms-21-04608-s001.zip › Figure S3.tif]

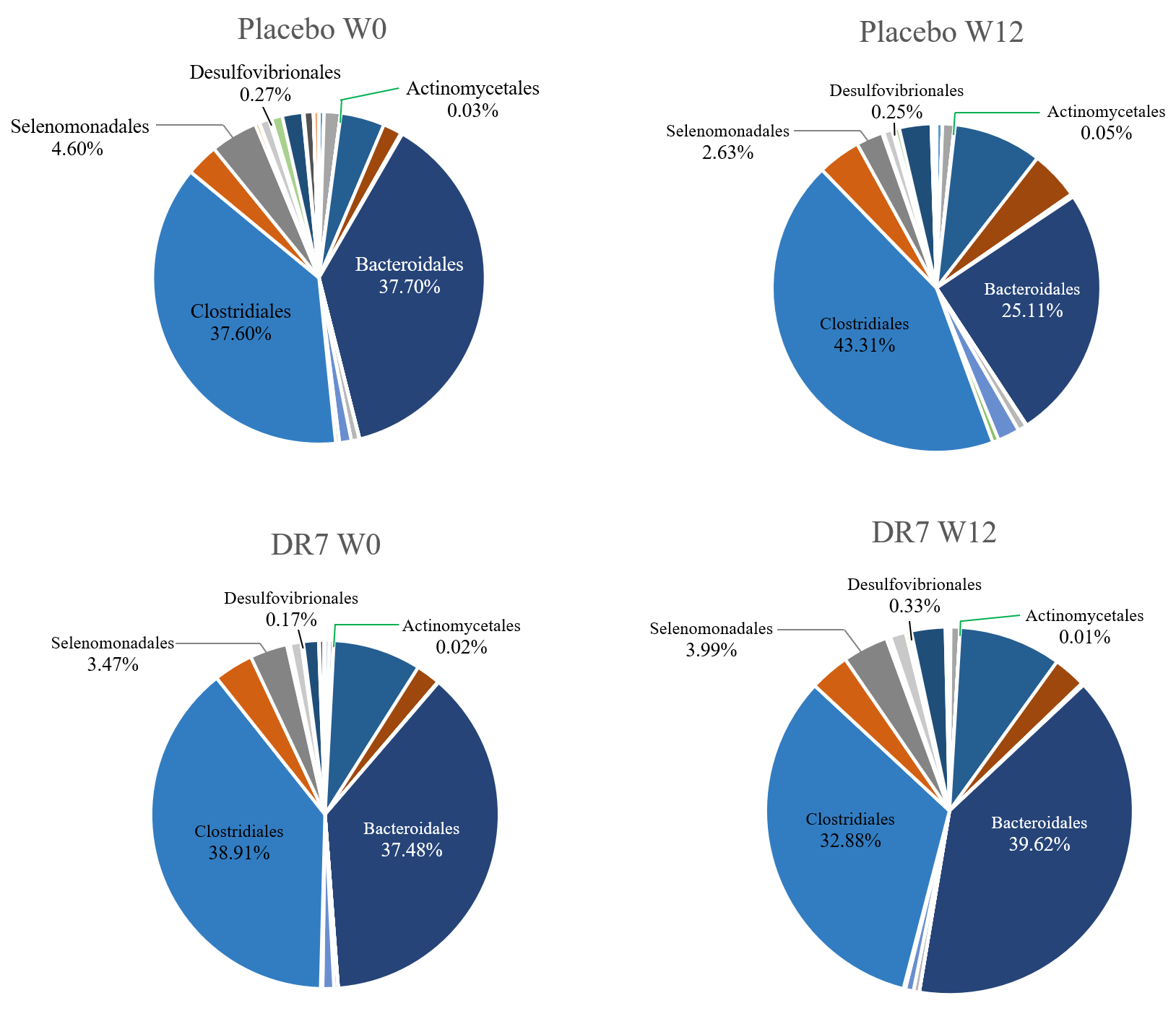

Supplement: Supplementary file 1 [file ijms-21-04608-s001.zip › Figure S4.tif]

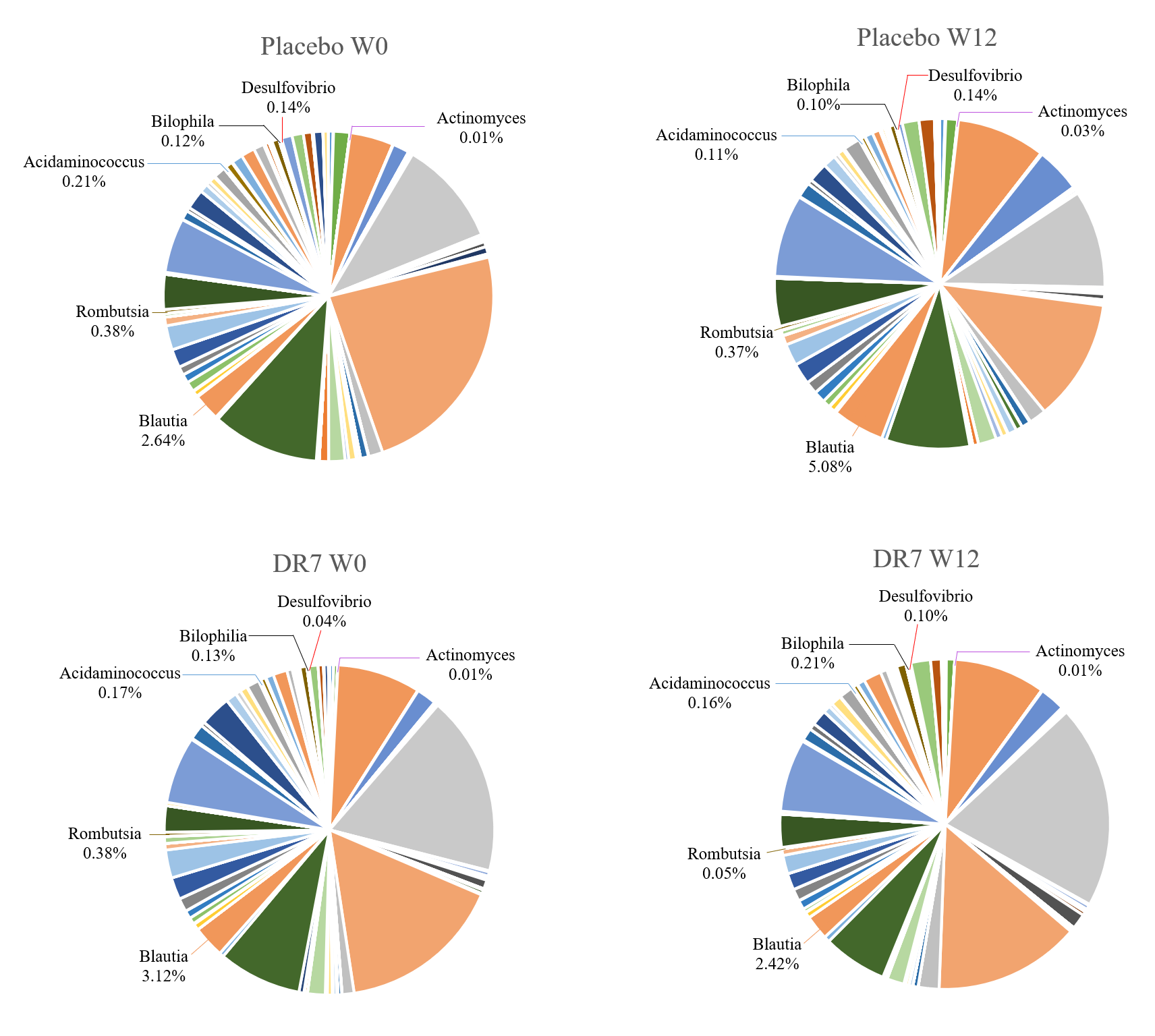

Supplement: Supplementary file 1 [file ijms-21-04608-s001.zip › Figure S5.tif]
